# Arterial and venous vascular complications in patients requiring peripheral venoarterial extracorporeal membrane oxygenation

**DOI:** 10.3389/fmed.2022.960716

**Published:** 2022-07-28

**Authors:** Christoph Fisser, Corina Armbrüster, Clemens Wiest, Alois Philipp, Maik Foltan, Dirk Lunz, Karin Pfister, Roland Schneckenpointner, Christof Schmid, Lars S. Maier, Thomas Müller, Matthias Lubnow

**Affiliations:** ^1^Department of Internal Medicine II, University Hospital Regensburg, Regensburg, Germany; ^2^Department of Cardiothoracic Surgery, University Hospital Regensburg, Regensburg, Germany; ^3^Department of Anesthesiology, University Hospital Regensburg, Regensburg, Germany; ^4^Department of Vascular Surgery, University Hospital Regensburg, Regensburg, Germany

**Keywords:** ECMO, vascular complication, ischemia, thrombosis, decannulation, bleeding, NIRS, risk factor

## Abstract

**Introduction:**

The aim of this study was to investigate the prevalence of arterial and venous complications in patients requiring peripheral venoarterial extracorporeal membrane oxygenation (VA ECMO) and its risk factors at the time of cannulation and during extracorporeal membrane oxygenation (ECMO) support and to assess vascular complications in association with decannulation.

**Material and methods:**

Between January 2010 to January 2020, out of 1,030 eligible patients requiring VA-ECMO, 427 with analyzable vascular screening were included. Duplex sonography and/or CT scan after decannulation were used to screen for thrombosis and pulmonary embolism as well as arterial complications. Near-infrared spectrometry (NIRS) was established at the time of cannulation and was continuously monitored during the ECMO therapy.

**Results:**

The prevalence of venous complications was 27%. Thrombosis and pulmonary embolism were observed in 21 and 7% of patients, respectively. Pulmonary embolism was more frequently diagnosed in patients with thrombosis (22 vs. 3%, *p* < 0.001). In multivariate analysis, cannulation in the jugular vein was determined as a risk factor for venous thrombosis in contrast to the extent of anticoagulation. The prevalence of arterial complications was 37%, mainly ischemia followed by bleeding, dissection, and compartment syndrome. Vascular surgery was necessary for 19% of the patients, of whome 1% required major amputations. A distal perfusion cannula (DPC) was implanted at cannulation in 24% of patients and secondarily in 16% of patients after cannulation as required during ECMO support. In the multivariate analysis, risk factors for leg ischemia at the time of cannulation were elevated D-dimers, lower NIRS on the cannulated leg, and lack of a DPC. The best discriminative parameter was the difference in NIRS between the non-cannulated leg and the cannulated leg. In contrast, during ECMO support, only the lack of a DPC was associated with leg ischemia. A similar rate of complications associated with decannulation, mainly arterial thrombosis, ischemia, or bleeding, was seen with percutaneous and surgical approaches (18 vs. 17%, *p* = 0.295).

**Conclusion:**

Patients requiring VA ECMO should be routinely screened for vascular complications. The decision to insert a DPC should be evaluated individually. However, NIRS monitoring of the cannulated leg and the non-cannulated leg is essential to identify the legs at risk for critical ischemia. As complications associated with decannulation were equally distributed between percutaneous and surgical approaches, the applied method may be chosen according to local experience.

## Introduction

Despite technological improvements and increasing clinical experience with venoarterial extracorporeal membrane oxygenation (VA ECMO), significant complications arise during VA-ECMO therapy either due to the therapy itself or due to the complexity of the critically ill patient.

A frequent complication in venovenous (VV) ECMO is thromboembolism, affecting more than 50% of patients to various extents (1). In contrast to the extensive knowledge available on VV ECMO, remarkably little is known about venous complications such as thrombosis and pulmonary embolism in patients requiring VA ECMO. This lack of knowledge is even more surprising considering the fact that patients requiring VA ECMO are more severely ill than patients requiring VV ECMO (2). For instance, patients requiring VA ECMO more frequently present with liver failure, which may affect coagulation, and often need different anticoagulation strategies than patients with VV ECMO support (3).

A distinct feature of peripheral VA ECMO in comparison to VV ECMO is the cannulation of a major artery, which is frequently accompanied by arterial vascular complications such as critical limb ischemia (4). It is known that critical limb ischemia during VA ECMO is associated with increased demands on medical resources, lower quality of life, and poor outcomes (5). Further, arterial vascular complications, such as bleeding, dissection, compartment syndrome, or initially failed puncture, and complications in association with decannulation are seldomly reported and need to be more emphasized.

Therefore, the aim of this study was to investigate the prevalence of arterial and venous vascular complications in patients requiring peripheral venoarterial extracorporeal membrane oxygenation (VA ECMO) and its risk factors at the time of cannulation and during ECMO support and to assess the complications in association with decannulation.

## Materials and methods

### Study subjects

All consecutive adult patients supported with VA ECMO at the University Hospital Regensburg between January 2010 and January 2020 were eligible for this analysis. Patients who had received central VA ECMO cannulation or had died during the ECMO therapy were excluded because screening for vascular complications after decannulation was not conducted in these patients.

Indications for VA ECMO were the cardiogenic shock of different etiologies and extracorporeal cardiopulmonary resuscitation (eCPR).

The study was conducted according to the Declaration of Helsinki on Good Clinical Practice. The requirement of individual patient consent and the necessity of approval for the data report were waived by the local ethics committee (20-1710-104) because of the retrospective, anonymized study design and of the analysis of data exclusively collected during routine care.

Patient data such as demographics, biochemistry, hemodynamic parameters, resuscitation status, sequential organ failure assessment (SOFA), and computed tomography images were extracted from the electronic patient data management system. The preexisting vascular risk status was defined according to the diagnosis of cerebrovascular disease (CVD), peripheral artery disease (PAD), or coronary artery disease (CAD). Disseminated intravascular coagulopathy was defined as that stated in a previous study (1), with good neurologic outcome as a cerebral performance score (CPC) of 1 (good cerebral performance) or 2 (moderate disability) and poor neurologic outcome as a CPC of 3–5 (6). Survival was assessed at discharge from the hospital.

### Cannulation and decannulation technique and anticoagulation

In general, drainage cannulae were placed into the femoral vein and return cannulae into the femoral artery either on the same side (mainly during eCPR) or bilaterally. Adaptions were allowed according to the anatomy or the treating physician. The drainage and the return cannulae were implanted percutaneously *via* Seldinger’s technique by an experienced intensivist or in the operation room by a surgeon. A vascular ultrasound scan was carried out prior to cannulation when possible. The size of the arterial and venous cannulae was chosen as previously published according to ultrasound findings, the desired ECMO flow rate, and the patients’ physical dimensions (4). The position of the cannulae was checked with ultrasound and x-ray or CT scan. Patients without any previous therapeutic anticoagulation received a bolus of up to 5,000 IU unfractionated heparin for cannulation. The circuit design and components of cannulation are depicted in Supplementary Figure 1.

During ECMO support, we aimed for an activated partial thromboplastin time of 60 ± 5 s in accordance with current recommendations (3). Further details on anticoagulation have been previously published (1, 4) and are presented in Supplementary Table 1.

A distal wire-reinforced perfusion cannula (DPC, CruraSave femoral-Perfusion Set 7 Fr, Free life medical GmbH, Aachen, Germany) into the superficial femoral artery was not routinely inserted (Supplementary Figure 2). The placement of a DPC was usually made in the ICU in the case of clinical signs of reduced leg perfusion at the time of cannulation or during the ECMO therapy. The flow rate of the DPC was checked routinely every 8–24 h and more frequently in case of a decline in near-infrared spectrometry (NIRS) (4).

Decannulation was performed either percutaneously with manual compression at the bedside or by surgeons in the operating theater after discontinuation of anticoagulation for at least 4 h. After control of bleeding by manual compression and skin suture, an inflatable balloon tape system (SafeGuard; MeritEMEA, Limburg, The Netherlands) was attached for at least 24 h. The balloon device was stepwise deflated every 2–4 h until complete removal. Alternatively, a compression bandage was applied.

### Venous complications

Screening for venous thrombosis was conducted with duplex sonography within 3 days after decannulation. The diagnosis was made by specially trained physicians in the case of incompressible veins and absent or reduced blood flow as indicated in a previously study (1, 7). Obstruction of the venous lumen diameter of >50% was classified as major thrombosis (1, 7). Additionally, all available CT scans made for various clinical indications after decannulation during the hospital stay were analyzed for new events of pulmonary embolism and thrombosis (1). Additionally, we assessed other complications such as bleeding at the site of cannulation, atrial/ventricular perforation, and compartment syndrome that might occur in parallel to thromboembolic complications.

### Arterial complications

Risk factors for limb ischemia were collected from the initiation until the end of ECMO support. Regional oxygen saturation in both legs was continuously measured by NIRS (INVOS™ 5100C, Medtronic, Minneapolis, United States). For the analysis of the entire ECMO support, NIRS was documented only one time a day, i.e., in the morning. Acute desaturation in NIRS as a sign of deterioration of perfusion/ischemia immediately resulted in further diagnostic workup and intervention to prevent limb ischemia (e.g., placement of a DPC). In addition, clinical signs of arterial complications of the leg (pallor, hypothermia, or pulselessness) or bleeding were checked routinely every 2 h. Doppler ultrasonography of the dorsalis pedis artery and the posterior tibial artery was routinely performed every 8 h and more frequently in the case of suspected ischemia. If possible, preventive measures to reduce the risk of limb ischemia such as improvement of leg perfusion were applied by means of reduction of vasopressors, infusion of vasodilators, or use of inotropes. In the case of incipient limb ischemia of a cannulated leg without a primarily implanted DPC, a DPC was inserted with the guidance of ultrasound.

Based on previous publications, critical limb ischemia was defined as a decrease in NIRS by 25% compared to the contralateral leg, a decrease in absolute NIRS values below 40%, showing clinical signs of ischemia, or showing sonographic evidence of missing perfusion (4). Additionally, we assessed other complications such as arterial thrombosis, bleeding at the site of cannulation, arterial dissection, compartment syndrome, and vascular surgery. Moreover, physicians’ documentation (including surgical protocols) and nurses’ shift reports were screened for vascular complications.

### Miscellaneous complications and complications associated with decannulation

Complications that could not be assigned to either the venous or arterial vascular system with certainty were considered miscellaneous complications, which included bleeding at the site of cannulation, arteriovenous fistula, and initially failed puncture during cannulation. We also assessed complications in association with decannulation such as ischemia, compartment syndrome, pseudoaneurysm, and bleeding.

### Statistics

All quantitative data are expressed as median (interquartile range) and were compared with the Mann–Whitney-*U* test. Differences between the study groups were assessed with the Chi-squared test of independence for nominal variables or the Fisher’s exact test as needed. Univariate logistic regression models were conducted to identify risk factors for venous thrombosis or limb ischemia. For limb ischemia, one model included parameters at the time of cannulation and the other model included parameters assessed during the ECMO therapy. The multivariat logistic regression model was adjusted for alle factors with a *p* values of less than 0.1 in the univariate analysis. In addition, a multivariate model for biochemistries according to limb ischemia was calculated. The cutoff points for NIRS were identified by receiver operating characteristic (ROC) analysis using the Youden index. All reported *p*-values were two-sided, and a *p*-value of ≤ 0.05 was considered statistically significant. Data entry and calculation were done using Microsoft EXCEL365 ProPlus (Microsoft, Redmond, WA, United States) and IBM SPSS Statistic software version 25.0 (SPSS Inc., Chicago, IL, United States).

## Results

### Study population

**FIGURE 1:**
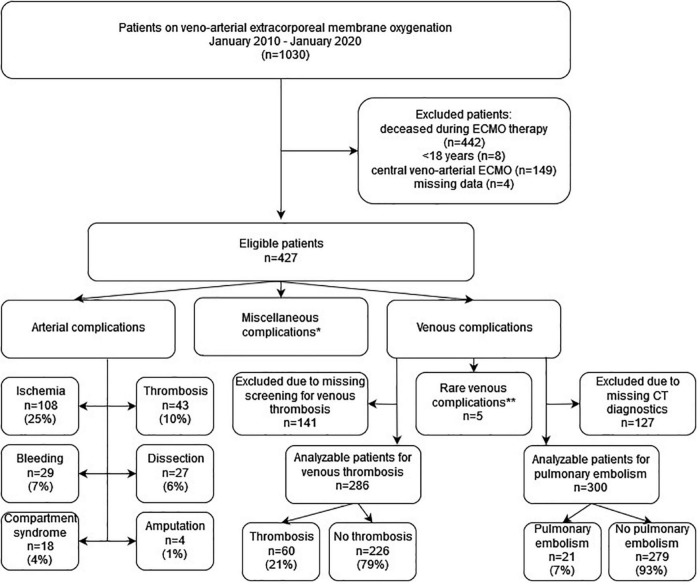
Flowchart of the observational study evaluating arterial and venous complications in survivors of venoarterial extracorporeal membrane oxygenation of the extracorporeal life support registry at Regensburg; ECMO, extracorporeal membrane oxygenation; CT, computer tomography; *miscellaneous complications included initially failed puncture (*n* = 24), bleeding (*n* = 20), and arteriovenous fistula (*n* = 1); **rare venous complications included bleeding (*n* = 2), right atrial/ventricular wall perforation (*n* = 2), and compartment syndrome of the leg with venous cannulation (*n* = 1).

From January 2010 to January 2020, 1,030 patients required VA ECMO at the University Hospital Regensburg, Germany, of whom 427 were eligible for the evaluation of venous and arterial complications (Figure 1). Patients had a median age of 59 [50; 68] years, a body mass index of 26.5 [24.2; 29.4] kg/m^2^, and were mainly men (73%); 64% of patients had arteriosclerosis and 22% had diabetes mellitus. Of the 427 patients, 161 (38%) of them received cannulation during eCPR (Supplementary Table 2). The SOFA score was 14 [12; 17], and 161 (38%) of the patients required renal replacement therapy. A DPC was implanted at cannulation in 101 (24%) patients and secondarily in 66 (16%) patients after cannulation as required during ECMO support. Baseline characteristics are summarized in Supplementary Table 3.

### Venous complications

**FIGURE 2:**
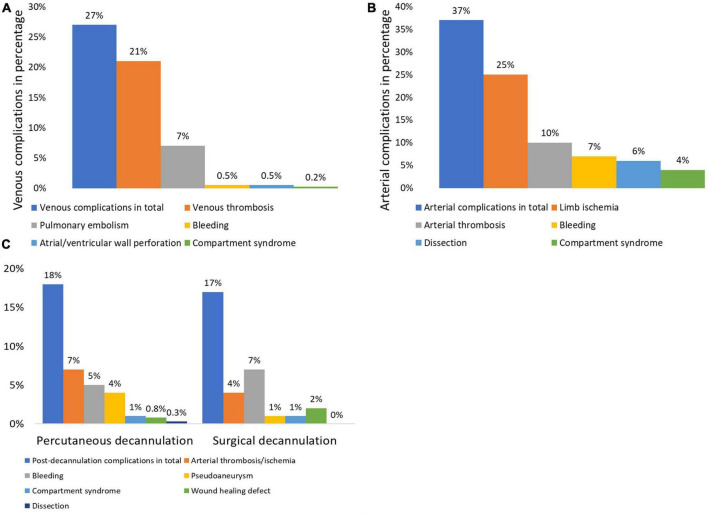
Prevalences of venous **(A)** and arterial **(B)** complications and **(C)** post-decannulation complications according to the type of decannulation in patients requiring venoarterial extracorporeal membrane oxygenation. Data are presented in percentages. All *p*-values comparing percutaneous vs. surgical decannulation are >0.05.

Screening for venous thrombosis was conducted in 67% (286/427) of patients. Venous thrombosis was prevalent in 21% (60/286) of patients and occlusion of the lumen diameter of > 50% was observed in 10% (29/286) of patients (Figure 2). After decannulation, thoracic CT scans with contrast dye were performed in 70% (300/427) of patients, of whom 7% (21/300) had pulmonary embolism. Pulmonary embolism was more frequently observed in those with thrombosis than those without thrombosis (22% [11/51] vs. 3% [5/168], *p* < 0.001). Overall, venous complications in patients undergoing screening for both pulmonary embolism and thrombosis were seen in 27% (60/219) of patients, including thromboembolic events in 26% (56/219) of them and other venous complications, such as bleeding at cannulation site (*n* = 2), right atrial/ventricular wall perforation (*n* = 2), and compartment syndrome of a leg with bilateral cannulation as a consequence of venous congestion (*n* = 1). Patients may have developed more than one complication.

### Risk factors for venous thrombosis

Risk factors for venous thrombosis were cannulation in the jugular vein in comparison to the femoral vein and the use of a small-sized cannula for venous drainage, but the latter did not occur after correcting for body surface area (Table 1 and Supplementary Table 4). Venous thrombosis was associated neither with unilateral arterial and venous cannulations nor with biochemistries (Table 2).

**TABLE 1:** Patient characteristics at cannulation with regard to venous thrombosis.

	*N*	Venous thrombosis *N* = 60	*N*	No venous thrombosis *N* = 226	*P*-value
Age, years	60	57 [48; 68]	226	59 [50; 68]	0.455
Sex, men	60	40 (67%)	226	161 (71%)	0.491
BMI, kg/m^2^	60	26.2 [23.6; 29.4]	226	26.8 [24.2; 29.4]	0.734
Diabetes mellitus	60	12 (20%)	226	49 (22%)	0.777
Disseminated intravascular coagulation	52	4 (8%)	185	24 (13%)	0.297
History of malignancy	60	5 (8%)	223	29 (13%)	0.323
Immunosuppression	60	2 (3%)	226	15 (7%)	0.539
Resuscitation pre ECMO	60	41 (68%)	226	150 (66%)	0.774
SOFA	48	15 [13; 17]	180	14 [12; 17]	0.259
Days on ECMO	60	4 [3; 7]	226	4 [3; 6]	0.572
Renal replacement therapy	60	29 (48%)	226	81 (36%)	0.077
Size of arterial cannula, French	60	15 [15; 17]	226	15 [15; 17]	0.276
Size of venous drainage cannula, French	60	21 [21; 21]	226	21 [21; 23]	**0.036**
Site of venous drainage cannula	60		226		**0.003**
Jugular		6 (10%)		3 (1%)	
Femoral		54 (90%)		223 (99%)	
Arterial and venous cannula ipsilateral	57	24 (42%)	219	100 (46%)	0.631
APTT, s	59	56 (38; 103)	215	51 (37; 115)	0.417
D-dimer, mg/L	53	12 (3; 24)	188	9 (3; 25)	0.412
INR	50	1.4 (1.2; 2.0)	195	1.4 (1.1; 1.8)	0.374
Fibrinogen, mg/dL	53	260 (192; 513)	191	252 (159; 378)	0.101
Antithrombin III, %	52	57 (46; 67)	186	56 (42; 67)	0.504
Plasma free hemoglobin, mg/dL	46	195 (65; 434)	174	146 (59; 362)	0.480
Platelets, /nL	59	174 (136; 235)	218	188 (127; 259)	0.705

Data are presented as median [25th; 75th percentile] or frequencies, n (%). Significant p-values (p < 0.05) are marked in bold. N = 286. BMI, body mass index; ECMO, extracorporeal membrane oxygenation; SOFA, sequential organ failure assessment; APTT, activated partial thromboplastin time.

**TABLE 2:** Univariate and multivariate binary logistic regression models for venous thrombosis in survivors of venoarterial extracorporeal membrane oxygenation.

Variables	Unadjusted		Adjusted	
	OR (95% CI)	*P*-value	OR (95% CI)	*P*-value
Age, years	0.990 (0.970; 1.011)	0.357		
Sex, men	1.238 (0.673; 2.277)	0.491		
BMI, kg/m^2^	1.008 (0.964; 1.054)	0.717		
Diabetes mellitus	1.107 (0.546; 2.246)	0.778		
Disseminated intravascular coagulopathy	1.789 (0.592; 5.409)	0.303		
History of malignancy	1.644 (0.608; 4.448)	0.327		
Immunosuppression	2.062 (0.458; 9.274)	0.346		
Resuscitation before ECMO	0.915 (0.497; 1.683	0.915		
SOFA	1.059 (0.964; 1.163)	0.234		
Days on ECMO	1.028 (0.974; 1.086)	0.317		
Renal replacement therapy	1.675 (0.943; 2.975)	0.079	1.421 (0.777; 2.597)	0.254
Size of arterial cannula, French	0.862 (0.690; 1.077)	0.190		
Size of arterial cannula per body surface area, French/m^2^	0.906 (0.666; 1.233)	0.530		
Size of venous cannula, French	0.714 (0.526; 0.970)	**0.031*^a^***		
Size of venous cannula per body surface area, French/m^2^	0.896 (0.701; 1.144)	0.379		
Site of venous cannula, jugular	8.259 (2.002; 34.082)	**0.004**	7.187 (1.701; 30.370)	**0.007**
Arterial and venous cannula ipsilateral	1.155 (0.641; 2.083)	0.631		
APTT, seconds	0.995 (0.970; 1.020)	0.683		
D-dimer, mg/L	1.010 (0.985; 1.035)	0.450		
Fibrinogen, mg/dL	1.000 (0.998; 1.002)	0.687		
Antithrombin III, %	1.008 (0.990; 1.028)	0.381		
Plasma free hemoglobin, mg/dL	1.001 (0.998; 1.005)	0.379		
Platelets, /nL	0.995 (0.989; 1.001)	0.085	1.000 (0.997; 1.003)	0.825
International normalized ratio	0.612 (0.147; 2.553)	0.500		

All parameters including biochemistries were assessed at the time of cannulation. Significant p-values (p < 0.05) are marked in bold. OR, odds ratio; CI, confidence interval; BMI, body mass index; ECMO, extracorporeal membrane oxygenation; SOFA, sequential organ failure assessment; APTT, activated partial thromboplastin time.

^a^Not included in the model because it is not significant after correction for BMI.

### Arterial complications

Complications associated with arterial cannulation were seen in 37% (158/427) of patients during ECMO support. Patients may have developed more than one complication; thus, limb ischemia was diagnosed in 25% (108/427) of patients, arterial thrombosis in 10% (43/427), bleeding in 7% (29/427), arterial dissection in 6% (27/427), and compartment syndrome in 4% (18/427). As a consequence, vascular surgery was performed in 19% (82/427) of patients, of whom 1% (4/427) required major amputation.

### Risk factors for limb ischemia at time of cannulation

Patients with limb ischemia were more frequently resuscitated, less frequently received a DPC, had lower NIRS at the cannulated and higher absolute differences in NIRS between the cannulated leg and the non-cannulated leg at the time of cannulation, and had slightly higher ECMO flow rate. No differences between the groups with and without limb ischemia were seen according to unilateral compared to bilateral cannulation, cannula size, or vasopressor therapy (Table 3). At the time of cannulation, the statistically best predictive NIRS value for limb ischemia was 18% (sensitivity 93%, specificity 18%) on the cannulated leg (Supplementary Figure 3). When comparing the difference between the non-cannulated leg and the cannulated leg with regard to limb ischemia, an absolute NIRS difference of 17% resulted in a sensitivity of 82% and a specificity of 60% (Supplementary Figure 4). In multivariate analysis, lack of a DPC, lower NIRS in the cannulated leg, and elevated D-dimers were associated with limb ischemia (Table 4).

**TABLE 3:** Patient characteristics at the time of cannulation with regard to ischemia.

	*N*	Ischemia *N* = 108	*N*	No ischemia *N* = 319	*P*-value
Age, years	108	57 (49; 65)	319	60 (50; 69)	0.100
Sex, men	108	77 (71%)	319	233 (73%)	0.725
BMI, kg/m^2^	108	26.3 (23.5; 30.5)	319	26.5 (24.2; 29.4)	0.983
Diabetes mellitus	108	22 (20%)	319	71 (22%)	0.681
Vascular risk (PAD, CAD, CVD)	108	74 (69%)	319	199 (62%)	0.251
Resuscitation pre ECMO	108	82 (76%)	319	208 (65%)	**0.039**
SOFA	90	14 (12; 18)	241	14 (12; 17)	0.338
Renal replacement therapy	108	42 (39%)	319	122 (38%)	0.905
**Cannula specifics**					
Percutaneous cannulation	108	103 (95%)	318	301 (95%)	0.771
Initially failed puncture	108	13 (12%)	319	11 (3%)	**<0.001**
Size of arterial cannula, French*^a^*	108	16 (15; 17)	319	15 (15; 17)	0.221
Size of venous cannula, French	108	21 (21; 21)	319	21 (21; 23)	0.078
Drainage and return cannulae ipsilateral	100	47 (47%)	287	118 (41%)	0.305
Distal perfusion cannula *a priori*	103	13 (13%)	296	88 (30%)	**<0.001**
NIRS cannulated leg, %	33	35 (28; 51)	59	49 (35; 61)	**0.035**
NIRS non-cannulated leg, %	21	63 (55; 65)	33	65 (53; 72)	0.310
NIRS difference between non-cannulated and cannulated leg, %*^b^*	21	25 (20; 37)	33	9 (0; 23)	**0.015**
ECMO blood flow, L/min	99	3.0 (2.5; 3.6)	306	2.9 (2.3; 3.3)	**0.044**
Mean arterial pressure, mmHg	101	55 (40; 65)	308	55 (41; 65)	0.745
Norepinephrine, μg/kg/min	106	0.36 (0.18; 0.74)	314	0.30 (0.14; 0.65)	0.218
Epinephrine, μg/kg/min	106	0.14 (0.00; 0.32)	314	0.10 (0.00; 0.24)	0.409
**Chemistries**					
APTT, s	102	50 (37; 89)	310	54 (37; 105)	0.797
D-dimer, mg/L	84	13 (4; 32)	255	7 (2; 19)	**0.006**
International normalized ratio	77	1.40 (1.20; 1.90)	250	1.40 (1.20; 1.80)	0.676
Fibrinogen, mg/dL	81	247 (149; 381)	257	266 (189; 394)	0.299
Antithrombin III, %	78	51 (46; 60)	248	57 (43; 67)	0.181
Plasma free hemoglobin, mg/dL	70	216 (72; 460)	221	134 (57; 345)	**0.043**
Platelets, /nL	103	182 (125; 284)	311	180 (136; 243)	0.928

Data are presented as median [25th; 75th percentile] or frequencies, n (%). Significant p-values (p < 0.05) are marked in bold. N = 427.

^a^Further details are presented in Supplementary Table 5.

^b^Only in patients with elective cannulation. BMI, body mass index; PAD, peripheral artery disease; CAD, coronary artery disease; CVD, cerebrovascular disease; ECMO, extracorporeal membrane oxygenation; SOFA, sequential organ failure assessment; NIRS, continuous near-infrared spectrometry; APTT, activated partial thromboplastin time.

**TABLE 4:** Univariate and multivariate binary logistic regression models for limb ischemia at the time of cannulation in survivors of veno-arterial extracorporeal membrane oxygenation.

Variables	Unadjusted		Model I		Model II	
	OR (95% CI)	*P*-value	OR (95% CI)	*P*-value	OR (95% CI)	*P*-value
Age, years	0.989 (0.973; 1.006)	0.203				
Sex, men	0.917 (0.565; 1.489)	0.725				
Body mass index, kg/m^2^	1.000 (0.965; 1.037)	0.986				
Diabetes mellitus	1.119 (0.654; 1.916)	0.681				
Vascular risk	0.762 (0.479; 1.213)	0.252				
Resuscitation pre ECMO	1.683 (1.023; 2.768)	**0.040**	2.013 (0.693; 5.849)	0.199		
SOFA	1.052 (0.981; 1.127)	0.157				
**RRT**						
**Cannula specifics**						
Initially failed puncture	3.832 (1.662; 8.833)	**0.002**	0.733 (0.087; 6.185)	0.775		
Size of arterial cannula, French	1.053 (0.914; 1.214)	0.472				
Size of venous cannula, French	1.165 (0.947; 1.434)	0.149				
Drainage and return cannulae ipsilateral	0.787 (0.498; 1.244)	0.305				
No distal perfusion cannula *a priori*	0.341 (0.181; 0.643)	**0.001**	0.182 (0.053; 0.623)	**0.007**		
NIRS cannulated leg, %	0.974 (0.949; 1.000)	**0.049**	0.958 (0.926; 0.992)	**0.015**		
NIRS non-cannulated leg, %	0.989 (0.952; 1.027)	0.561				
NIRS difference between non-cannulated and cannulated leg*^a^*, %	1.035 (1.000; 1.071)	0.053				
ECMO blood flow, L/min	1.427 (1.044; 1.949)	**0.026**	0.578 (0.248; 1.349)	0.205		
Lactate, mg/dL	1.003 (0.999; 1.008)	0.125				
Mean arterial pressure, mmHg	0.998 (0.984; 1.013)	0.824				
Norepinephrine, μg/kg/min	1.022 (0.829; 1.258)	0.841				
Epinephrine, μg/kg/min	1.444 (0.878; 2.372)	0.148				
**Chemistries**						
APTT, s	0.999 (0.992; 1.005)	0.639				
D-dimer, s	1.026 (1.007; 1.046)	**0.008**			1.023 (1.001; 1.046)	**0.038**
International normalized ratio	0.880 (0.659; 1.175)	0.386				
Fibrinogen, mg/dL	0.999 (0.998; 1.001)	0.398				
Antithrombin III, %	0.989 (0.976; 1.003)	0.114				
Plasma free hemoglobin, mg/dL	1.001 (1.000; 1.001)	**0.046**			1.001 (1.000; 1.001)	0.146
Platelets, /nL	1.000 (0.998; 1.002)	0.819				

All parameters including biochemistries were assessed at the time of cannulation. Significant p-values (p < 0.05) are marked in bold. OR, odds ratio; CI, confidence interval; ECMO, extracorporeal membrane oxygenation; RRT, renal replacement therapy; SOFA, sequential organ failure assessment; NIRS, continuous near-infrared spectrometry; APTT, activated partial thromboplastin time.

^a^Not included in the multivariate analysis due to over-adjustment with the NIRS cannulated leg.

### Risk factors for limb ischemia during extracorporeal membrane oxygenation support

During the entire ECMO support, patients with limb ischemia had lower median activated partial thromboplastin time (aPTT) and median NIRS values in the non-cannulated leg. Additional biochemistries, NIRS values, and vasopressors during ECMO support are provided in Supplementary Table 6. NIRS of the non-cannulated leg was associated with limb ischemia in the univariate analysis, but not in the multivariate analysis. The only factor that was associated with limb ischemia in the multivariate analysis was the lack of a DPC (Table 5).

**TABLE 5:** Univariate and multivariate binary logistic regression models for limb ischemia over the entire duration of ECMO support in survivors of venoarterial extracorporeal membrane oxygenation.

Variables	Unadjusted		Model I	
	OR (95% CI)	*P*-value	OR (95% CI)	*P*-value
Days on ECMO	0.988 (0.964; 1.033)	0.918		
APTT, s	0.988 (0.969; 1.007)	0.201		
D-dimer, mg/L	1.011 (0.991; 1.031)	0.294		
International normalized ratio	1.395 (0.572; 3.402)	0.465		
Fibrinogen, mg/dL	0.999 (0.997; 1.001)	0.222		
Antithrombin III, %	0.994 (0.980; 1.010)	0.466		
Plasma free hemoglobin, mg/dL	1.000 (0.998; 1.003)	0.698		
Platelets, /nL	0.999 (0.995; 1.004)	0.807		
NIRS cannulated leg, %*^a^*	0.999 (0.968; 1.031)	0.960		
NIRS non-cannulated leg, %*^a^*	0.964 (0.932; 0.996)	**0.027**	0.970 (0.925; 1.016)	0.199
Norepinephrine, μg/kg/min	4.023 (0.345; 46.978)	0.267		
Epinephrine, μg/kg/min	1.221 (0.021; 71.658)	0.924		
Subsequent implantation of distal perfusion cannula*^b^*	48.558 (21.695; 108.682)	**<0.001**	59.540 (19.756; 179.446)	**<0.001**

All parameters but distal perfusion cannula are depicted as median values during the ECMO therapy.

^a^NIRS was measured continuously but recorded for the study one time daily.

^b^Distal perfusion cannula (DPC) were not prophylactically used in each ECMO cannulation, but these patients received a DPC after cannulation in the course of ECMO support. Significant p-values (p < 0.05) are marked in bold. OR, odds ratio; CI, confidence interval; ECMO, extracorporeal membrane oxygenation; APTT, activated partial thromboplastin time; NIRS, continuous near-infrared spectrometry.

### Miscellaneous complications and complications associated with decannulation

Patients requiring a DPC at any time of ECMO treatment were more often diagnosed with limb ischemia, arterial thrombosis, and dissection and were in need of more packed red blood cells per day on ECMO support (Supplementary Table 7).

Initially failed puncture was seen in 6% (24/427), bleeding in 5% (20/427), and arteriovenous fistula in 0.2% (1/427). Complications in association with decannulation were observed in 17% (72/427) of patients. The most common decannulation complications were arterial thrombosis or ischemia, bleeding, and pseudoaneurysm, which were similarly distributed between percutaneous and surgical decannulation approaches (Table 6).

**TABLE 6:** Complications associated with decannulation in survivors of venoarterial extracorporeal membrane oxygenation.

	Total *N* = 427*	Percutaneous decannulation *N* = 264	Surgical decannulation *N* = 148	*P*-value
Cumulative complication rate	72 (17%)	47 (18%)	25 (17%)	0.295
Arterial thrombosis/ischemia*^a^*	24 (6%)	18 (7%)	6 (4%)	0.220
Bleeding	22 (5%)	12 (5%)	10 (7%)	0.205
Pseudoaneurysm*^b^*	13 (3%)	11 (4%)	2 (1%)	0.196
Compartment syndrome	5 (1%)	3 (1%)	2 (1%)	1.000
Dissection	1 (0.2%)	1 (0.3%)	0 (0%)	1.000
Wound healing defect	5 (1%)	2 (0.8%)	3 (2%)	0.334

Data are presented as frequencies, n (%). Patients may develop more than one complication.

*Missing information on type of decannulation in N = 15.

^a^Includes occlusion of the superficial femoral and common femoral artery and more distal arterial thrombosis.

^b^Includes one ateriovenous fistula.

### Outcome

After decannulation, 71% (302/427) of patients were discharged from the hospital with good neurological outcome in 76% (229/302) of them. Survival and good neurologic outcome were lower in those with limb ischemia than in those without limb ischemia (60% [65/108] vs. 74% [237/319], *p* = 0.005; 67% [42/63] vs. 80% [187/233], *p* = 0.022; missing data of CPC in *n* = 6).

## Discussion

This study provides novel insights into arterial and venous vascular complications in patients requiring and surviving VA ECMO. First, the prevalence of venous thrombosis was 21% and that of pulmonary embolism was 7%. The risk factor for venous thrombosis was venous jugular cannulation. Second, arterial complications were observed in 37% of patients. Risk factors for ischemia at the time of cannulation were lower NIRS of the cannulated leg and lack of a DPC. A difference of an absolute 17% in NIRS values between the non-cannulated leg and the cannulated leg showed the best predictive value for limb ischemia. Vascular complications associated with decannulation were observed in 17% of patients and were similarly distributed between percutaneous or surgical decannulation approaches.

### Prevalence and risk factors for venous thromboembolism in survivors of venoarterial extracorporeal membrane oxygenation

Data on venous complications in patients requiring VA ECMO are scarce (8, 9). In an autopsy study in postcardiotomy patients by Rastan et al. (9) (*n* = 78), a slightly higher prevalence of venous thrombosis (32%) and pulmonary embolism (15%) was reported in comparison to 21 and 7% in the current study. Compared to previous data from our VV ECMO cohort, the prevalence of venous thrombosis in the current study with VA ECMO was considerably lower (1). Reasons for these lower rates might result from the different patient populations with less time on ECMO support, the single venous ECMO cannula access, and the higher aPTT target levels. Nevertheless, it is important to emphasize that pulmonary embolism was observed more than seven times more frequently in those with thrombosis than in those without thrombosis. These findings support the need for systematic post-decannulation ultrasound screening in patients requiring VA ECMO.

In line with the aforementioned studies (1, 9), venous thrombosis was more prevalent in the jugular cannulated than in the femoral cannulated veins. Interestingly, no difference in the rate of thrombosis was seen in bilateral compared to unilateral cannulation. Unexpectedly, the use of a small-sized venous cannula was associated with venous thrombosis. However, after correcting for body surface, the use of a small-sized venous cannula positively correlated with the diameter of the peripheral veins (10), and no association was observed anymore.

### Prevalence and risk factors for arterial limb ischemia in patients requiring venoarterial extracorporeal membrane oxygenation

Frequencies of arterial complications, mainly limb ischemia, range from 2 to 52% due to different cannulation techniques and definitions of limb ischemia (11). In the current study, limb ischemia occurred in 25% of the patients and (12) was associated with mortality, as indicated in other studies. Therefore, to improve outcomes in patients on peripheral femoral VA ECMO, it is essential to detect patients at risk for limb ischemia at the time of cannulation and during the entire ECMO support. We identified lower NIRS values at the time of cannulation as an independent risk factor for the development of limb ischemia. However, the difference in NIRS values between the non-cannulated leg and the cannulated leg for limb ischemia at the time of cannulation was the best predictive value, eventually embedding the general hemodynamic status and the local perfusion of the arterially cannulated leg. Moreover, lack of a DPC, as reported by Tanaka et al. (13), and elevated D-dimers were associated with limb ischemia in contrast to unilateral compared to bilateral cannulation.

Over the entire ECMO support, only the lack of a DPC was associated with limb ischemia but not NIRS. The latter result seems to be in contrast to some smaller studies (each *n* < 65) (14–16); however, it can be explained by the usage of median values over the entire ECMO run without the inclusion of event-driven drops in NIRS values in case of acute ischemia. Thus, we believe NIRS monitoring to be essential to detect limb ischemia early to take immediate action to avoid severe complications.

### Miscellaneous complications and complications associated with decannulation

Notably, only 24% of patients required a DPC during cannulation and 16% of them after cannulation, which is in contrast to other studies with substantially higher (>90%) DPC placement rates at the beginning of ECMO support (17). Despite that, our rate of major amputations was lower compared to other studies (17–19). In addition, it has to be taken into account that DPC might be accompanied by vascular complications itself and more bleeding due to multiple unsuccessful sticks, dislodgement, or kinking. Therefore, the decision for DPC placement should be made on an individual basis.

Complications other than limb ischemia are seldomly reported or grouped together (17, 19, 20). In particular, initially failed puncture at the site of cannulation has never been reported as a risk factor for limb ischemia. This complication is of interest because it may be avoidable by accurate identification of the vessels by applying ultrasound during cannulation. However, this association was not robust in the multivariate analysis.

Data on vascular complications in association with decannulation are lacking besides a case series comparing a percutaneous closure device with surgical decannulation reporting higher complication rates after the surgical approach (21, 22). The best method for decannulation either surgically or percutaneously is still unclear (23). In this analysis, decannulation was conducted percutaneously in more than 60% of patients with similar complication rates than with surgical decannulation. However, it is noteworthy that, irrespective of the decannulation method, limb ischemia, bleeding, and pseudoaneurysm were observed in 6, 5, and 3%, respectively. In consequence, all patients should routinely be screened after decannulation for potential complications (23).

### Strength and limitations

The strength of the current study is the systematic screening for both arterial *and* venous complications and the inclusion of rare complications as well as of complications associated with decannulation. The exact timing of complications was limited by the applied screening strategy. The classification of risk factors for limb ischemia into those risks at the time of cannulation and risks during the entire ECMO support may help clinicians to identify patients with a high risk of limb ischemia at cannulation and during the ECMO therapy.

Due to the retrospective design, underreporting of complications may have occurred. The multivariate analysis was only carried out for the most frequent events. For the analysis over the entire ECMO support, NIRS was documented only one time per day. Patients with central VA ECMO and those with the application of a percutaneous closure device for decannulation were not included in this analysis.

## Conclusion

Patients with VA-ECMO should be routinely screened for vascular complications, and necessary anticoagulation should be provided as arterial and venous complications under peripheral VA ECMO are frequently seen. A drainage cannula in the jugular vein was a risk factor for venous thrombosis and should be avoided, if possible. The placement of a DPC was necessary in 40% of patients and the decision to insert a DPC should be evaluated individually. NIRS monitoring of the cannulated and the non-cannulated leg at the time of cannulation and during ECMO is essential to identify limbs at risk for critical ischemia to allow immediate action to avoid severe complications. As complications associated with either percutaneous or surgical decannulation were equally distributed, the applied method may be chosen according to local experience and patient-specific factors.

## Data availability statement

The original contributions presented in this study are included in the article/Supplementary material, further inquiries can be directed to the corresponding author.

## Ethics statement

The studies involving human participants were reviewed and approved by University of Regensburg, Germany, 20-1710-104. Written informed consent for participation was not required for this study in accordance with the national legislation and the institutional requirements.

## Author contributions

CF and ML were responsible for the conception, hypotheses delineation, and the design of the study, for the acquisition of data, the analysis and interpretation of this information, and for writing the article and its revision prior to submission. CF, ML, and CA were responsible for drafting the manuscript and were involved in the acquisition of data, the analysis and interpretation of this information, and the critical revision of the article prior to submission. CW, RS, MF, AP, DL, KP, CS, and TM were involved in the acquisition of data, the analysis and interpretation of results, and the critical revision of the article prior to submission. All authors contributed to the article and approved the submitted version.

## Abbreviations

CAD: coronary artery disease
CPC: cerebral performance category
CVD: cerebrovascular disease
DPC: distal perfusion cannula
ECMO: extracorporeal membrane oxygenation
ECPR: extracorporeal cardiopulmonary resuscitation
IU: international units
PAD: peripheral artery disease
ROC: receiver operating curve
SOFA: sequential organ failure assessment
VA: veno-arterial
VV: veno-venous.
