# Supplementary material for: Arterial and venous vascular complications in patients requiring peripheral venoarterial extracorporeal membrane oxygenation
Source: Front Med (Lausanne). 2022 Jul 28;9:960716. doi: 10.3389/fmed.2022.960716 (PMC9365977; doi:10.3389/fmed.2022.960716)
Supplement: Supplementary file 1 [file Table_7.DOCX]

**Supplemental material:**

This appendix has been provided by the authors to give readers additional information about the study.

Supplement to:

**Arterial and venous vascular complications in patients requiring peripheral veno-arterial extracorporeal membrane oxygenation**

Christoph Fisser^1^; Corina Armbrüster^1^; Clemens Wiest^1^; Alois Philipp^2^; Maik Foltan^2^; Dirk Lunz^3^; Karin Pfister^4^; Roland Schneckenpointner^1^; Christof Schmid^2^; Lars S. Maier^1^; Thomas Müller^1^; Matthias Lubnow^1^

^1^ Department of Internal Medicine II, University Hospital Regensburg, Regensburg, Germany

^2^ Department of Cardiothoracic Surgery, University Medical Center Regensburg, Regensburg, Germany

^3^ Department of Anesthesiology, University Medical Center Regensburg, Regensburg, Germany

^4^ Department of Vascular Surgery, University Medical Center Regensburg, Regensburg, Germany

**Material and methods**

*Oxygenators and Cannulae*

The type of oxygenator (Cardiohelp 5.0, 7.0, IR, ELS, PALP, PLS; Quadrox-iD Ped, Maquet, Germany and Hilite LT7000, ILA active; Novalung, Heilbronn, Germany and ECC.O5; Sorin, Italy) was chosen by availability and patient-specific needs.

In general, 21 French (Fr) cannula, 55 cm length was used as drainage and 15 Fr/15 cm as return cannula (HLS, Getinge Cardiovascular, Rastatt, Germany). However, adaptions were allowed according to availability and patient-specific needs.

Figure S1A: Circuit design


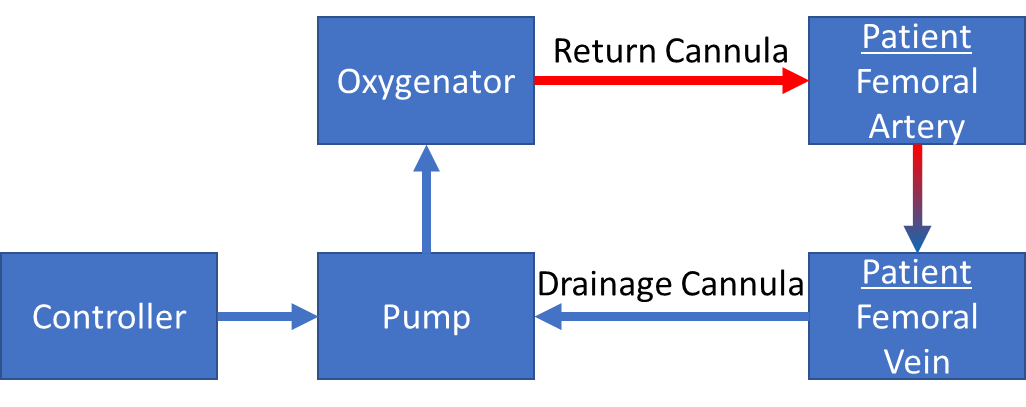


Figure S1B: Clinical setting of veno-arterial extracorporeal membrane oxygenation and CT-Abdomen.

The drainage cannula is located in the right femoral vein, and the return canula in the left femoral artery with additional distal perfusion cannula with Near-Infrared Spectrometry monitoring and CT-Abdomen of the same patient with drainage cannula (blue arrow) and return cannula (red arrow).

*Anticoagulation*

The flow-rate of unfractionated heparin was titrated and adapted to the target range 60±5 seconds according to former recommendations ((1); table S1). APTT levels were checked every 4-6 hours, until target range was achieved. In special situations such as coagulation disorders or thrombocytopenia adjustments of heparin dosage were allowed individually.

Anticoagulation parameters were checked prior to ECMO, 2 hours after implantation and at least once per day on ECMO, and at least for 2 days after decannulation, usually daily until discharge from ICU. In the case of bleeding, anticoagulation parameters were checked again, and anticoagulation was adapted to the extent of bleeding; in severe cases heparin was stopped and, tranexamic acid, fresh frozen plasma, platelets, fibrinogen, and prothrombin concentrates were administered as required.

Anticoagulation with target aPTT of 60±5 seconds is continued until screening for venous thrombotic events after decannulation was performed. In the case of a thrombotic event, we aim for an aPTT target of 60-80 seconds, if no other contraindication for anticoagulation is present. If no thrombotic event is observed, anticoagulation is switched to prophylactic dose if not otherwise indicated.

Figure S2: Image of wire-reinforced distal perfusion cannula


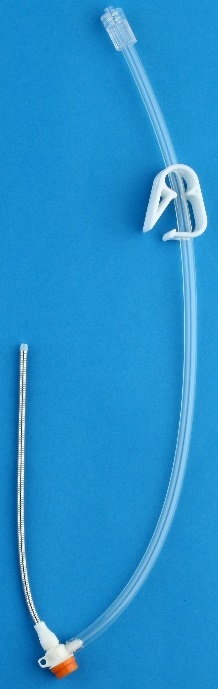


Table S1: Anticoagulation protocol adapted according to Raschke et al. (1)

| Initial dose | 80 units/kg bolus, then 18 units/kg/h |
| --- | --- |
| aPTT, < 35 s | 80 units/kg bolus, then increase 4 units/kg/h |
| aPTT, 35-45 s | 40 units/kg bolus, then increase 2 units/kg/h |
| aPTT, 46-55 s | Increase infusion rate by 2 units/kg/h |
| aPTT, 56-70 s | No change |
| aPTT, 71-90 s | Decrease infusion rate by 2 units/kg/h |
| aPTT, > 90 s | Hold infusion 1 h, then decrease infusion rate by 3 units/kg/h |

aPTT: activated partial thromboplastin time

Table S2: Indication for veno-arterial extracorporeal membrane oxygenation

| Indication | N=427 |
| --- | --- |
| Low cardiac output | 118 (28%) |
| Cardiopulmonary resuscitation less than 12 hours before VA ECMO | 100 (23%) |
| Extracorporeal cardiopulmonary resuscitation | 161 (38%) |
| Cardiopulmonary resuscitation during interventional procedure | 29 (7%) |
| No weaning from extracorporeal bypass during cardio-thoracic surgery | 19 (4%) |

Data are presented as frequencies in n (%). VA ECMO: veno-arterial extracorporeal membrane oxygenation.

Table S3: Additional baseline parameters in the context of cannulation

| Variable | N |  |
| --- | --- | --- |
| Vascular risk | 427 | 273 (64%) |
| Resuscitation before ECMO | 427 | 290 (68%) |
| Days on ECMO | 427 | 4 [3; 6] |
| Size of arterial cannula, French | 427 | 15 [15; 17] |
| Size of venous cannula, French | 427 | 21 [21; 23] |
| History of malignancy | 424 | 50 (12%) |
| Immunosuppression | 427 | 19 (4%) |
| Disseminated intravascular coagulation | 327 | 40 (9%) |
| pO2/FiO2, mmHg | 329 | 98 [68; 195] |
| pCO2, mmHg | 328 | 46 [38; 56] |
| Base excess, mmol/L | 357 | -8 [-13; -4] |
| pH | 336 | 7.23 [7.13; 7.34] |
| Lactate, mg/dL | 420 | 74 [38; 107] |
| Mean arterial pressure, mmHg | 409 | 55 [40; 65] |
| Norepinephrine, µg/kg/min | 420 | 0.31 [0.15; 0.67] |
| Epinephrine, µg/kg/min | 420 | 0.11 [0.00; 0.26] |
| aPTT, s | 412 | 54 [37; 99] |
| D-Dimer, mg/L | 339 | 9 [3; 23] |
| Fibrinogen, mg/dL | 338 | 266 [173; 388] |
| Antithrombin III, % | 326 | 57 [42; 67] |
| Plasma-free hemoglobin, mg/L | 291 | 148 [59; 363] |
| International normalized ratio | 327 | 1.40 [1.20; 1.80] |
| Platelets, 10^9^/L | 414 | 181 [128; 244] |

Data are presented as median [25^th^; 75^th^ percentile] or frequencies, n (%). Significant p values (p < 0.05) are marked in bold. Vascular risks include cerebrovascular disease, peripheral artery disease and coronary artery disease. BMI: body mass index; SOFA: sequential organ failure assessment; RRT: renal replacement therapy; ECMO: extracorporeal membrane oxygenation; aPTT: activated partial thromboplastin time. All parameters were assessed in the context of ECMO cannulation.

Table S4: Size of venous cannula in the context of venous thrombosis

| Variables | Venous thrombosis  N=60 | No venous thrombosis  N=226 | p-value |
| --- | --- | --- | --- |
| Size of venous cannula, French |  |  | 0.158 |
| 19 | 2 (3%) | 3 (1%) |  |
| 21 | 46 (77%) | 149 (66%) |  |
| 23 | 12 (20%) | 70 (31%) |  |
| 25 | 0 (0%) | 4 (2%) |  |

Data are presented as frequencies, n (%). The 15 French cannula was used as return cannula in veno-arterial-venous extracorporeal membrane oxygenation. Values are rounded.

Table S5: Size of arterial cannula in the context of limb ischemia

| Variables | No limb ischemia  N=319^a^ | | | p-value no DPC vs DPC | Limb ischemia  N=108^b^ | | | p-value no DPC vs DPC | p-value no limb ischemia vs. limb ischemia |
| --- | --- | --- | --- | --- | --- | --- | --- | --- | --- |
| Size of arterial cannula, French | Total | No DPC  N=199 | DPC  N=96 | 0.590 | Total | No DPC N=30* | DPC N=71 | **0.031** | 0.652 |
| 13 | 3 (1%) | 2 (1%) | 1 (1%) |  | 1 (1%) | 0 (0%) | 1 (1%) |  |  |
| 15 | 180 (56%) | 118 (59%) | 49 (51%) |  | 53 (49%) | 19 (63%) | 31 (44%) |  |  |
| 17 | 119 (37%) | 69 (35%) | 42 (44%) |  | 48 (44%) | 8 (27%) | 37 (52%) |  |  |
| 19 | 7 (2%) | 4 (2%) | 3 (3%) |  | 4 (4%) | 1 (3%) | 2 (3%) |  |  |
| 21 | 6 (2%) | 4 (2%) | 1 (1%) |  | 1 (1%) | 1 (3%) | 0 (0%) |  |  |
| 23 | 4 (1%) | 2 (1%) | 0 (0%) |  | 1 (1%) | 1 (3%) | 0 (0%) |  |  |

Data are presented as frequencies, n (%). DPC: distal perfusion cannula at any time during therapy with extracorporeal membrane oxygenation; *Patients did not receive a DPC, for instance because of hypoxic brain damage, multi-organ failure, or no chance of functional recovery/heart transplant/left ventricular assist device; missing data for DPC ^a^ n=24, ^b^ n=7; significant p values (p < 0.05) are marked in bold. Values are rounded.

Table S6: Additional characteristics over the entire duration of extracorporeal membrane oxygeantion support

|  | Ischemia  N=108 | No ischemia  N=319 | p-value |
| --- | --- | --- | --- |
| Days on ECMO | 4 (2; 7) | 4 (3; 6) | 0.413 |
| APTT, s | 49 [44; 56] | 52 [46; 59] | **0.031** |
| D-Dimer, mg/L | 9 [4; 20] | 8 [4; 18] | 0.311 |
| INR | 1.25 [1.14; 1.40] | 1.20 [1.10; 1.40] | 0.367 |
| Fibrinogen, mg/dL | 342 [264; 472] | 372 [303; 478] | 0.071 |
| Antithrombin III, % | 60 [48; 71] | 60 [52; 70] | 0.623 |
| Plasma free hemoglobin, mg/dL | 52 [37; 74] | 51 [39; 83] | 0.474 |
| Platelets, /nL | 99 [70; 138] | 98 [65; 139] | 0.882 |
| NIRS cannulated leg, rSO_2_ % | 71 (64; 77) | 72 (63; 78) | 0.910 |
| NIRS non-cannulated leg, rSO_2_ % | 68 (60; 76) | 72 (66; 79) | **0.021** |
| Norepinephrine, µg/kg/min | 0.02 [0.00; 0.07] | 0.02 [0.00; 0.07] | 0.700 |
| Epinephrine, µg/kg/min | 0.03 [0.00; 0.06] | 0.03 [0.00; 0.07] | 0.973 |
| Subsequent implantation of distal perfusion cannula^a^ | 58 (54%) | 8 (3%) | **<0.001** |

Data are presented as median [25^th^; 75^th^ percentile]. N=427. ECMO: extracorporeal membrane oxygenation; APTT: activated partial prothrombin time, INR: international normalized ratio, NIRS: near-infrared spectroscopy. NIRS values were assessed once daily every day during the ECMO support. ^a^ Distal perfusion cannulae (DPC) were not prophylactically implanted in each ECMO cannulation, but these patients received a DPC after cannulation in the course of ECMO support to decrease the NIRS value. rSO_2_: regional oxygen saturation. Significant p values (p < 0.05) are marked in bold. Variables are depicted as median of the entire ECMO duration.

Table S7: Complications according to presence of distal perfusion cannula

|  | No distal perfusion cannula  N=229 | Distal perfusion cannula  N=167 | p-value |
| --- | --- | --- | --- |
| Limb ischemia | 30 (13%) | 71 (43%) | **<0.001** |
| Compartment syndrome^a^ | 10 (5%) | 6 (4%) | 0.665 |
| Vascular surgery^b^ | 38 (18%) | 38 (23%) | 0.244 |
| Amputation^c^ | 1 (0.5%) | 3 (2%) | 0.319 |
| Dissection^d^ | 6 (3%) | 20 (12%) | **<0.001** |
| Bleeding^e^ | 27 (12%) | 20 (12%) | 0.866 |
| Intracranial bleeding^f^ | 9 (4%) | 7 (4%) | 0.917 |
| Mispuncture | 11 (5%) | 12 (7%) | 0.317 |
| Arterial thrombosis | 14 (6%) | 26 (16%) | **0.001** |
| Venous thrombosis^g^ | 30 (20%) | 28 (22%) | 0.689 |
| Pulmonary embolism^h^ | 8 (6%) | 8 (6%) | 0.857 |
| Packed red blood^i^ cells per day on ECMO | 0.40 [0.00; 1.00] | 0.67 [0.00; 1.36] | **0.040** |
| Fresh frozen plasma^j^ per day on ECMO | 0.00 [0.00; 0.20] | 0.00 [0.00; 0.73] | 0.146 |
| Platelet transfusion^i^ per day on ECMO | 0.00 [0.00; 0.22] | 0.00 [0.00; 0.33] | 0.077 |

Data are presented as median [25^th^; 75^th^ percentile] or frequencies, n (%). Significant p values (p < 0.05) marked in bold. N=427*. Distal perfusion cannula (DPC) at any time during the ECMO support. One patient can have more than one event. *missing information on DPC n=31

^a^missing information of compartment syndrome in no DPC n=8 and in DPC n=2; compartment syndrome due to arterial complication

^b^missing information of vascular surgery in no DPC n=14 and in DPC n=2

^c^missing information of amputation in no DPC n=11 and in DPC n=2

^d^missing information of dissection in no DPC n=23 and in DPC n=7

^e^missing information of bleeding in no DPC n=2 and in DPC n=0; arterial and venous bleeding.

^f^missing information of intracranial bleeding in no DPC n=7 and in DPC n=3

^g^missing information of venous thrombosis in no DPC n=82 and in DPC n=42; patients with screening for venous thrombosis n=286

^h^missing information of pulmonary embolism in no DPC n=86 and in DPC n=36; patients with screening for pulmonary embolism n=300

^i^missing information of packed red blood cells in no DPC n=11 and in DPC n=4

^j^missing information of fresh frozen plasma in no DPC n=2 and in DPC n=5

Figure S3: Receiver operating curve for INVOS pre cannulation


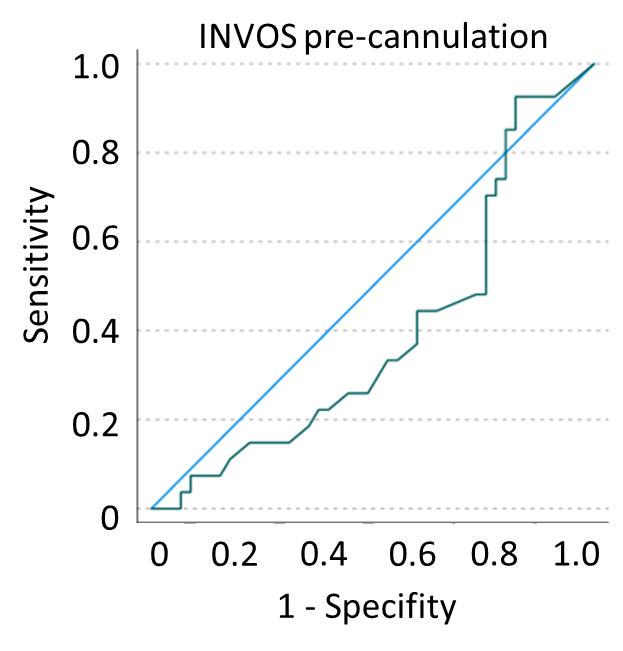


Figure S4: Receiver operating curve for INVOS difference between the non-cannulated and the cannulated leg


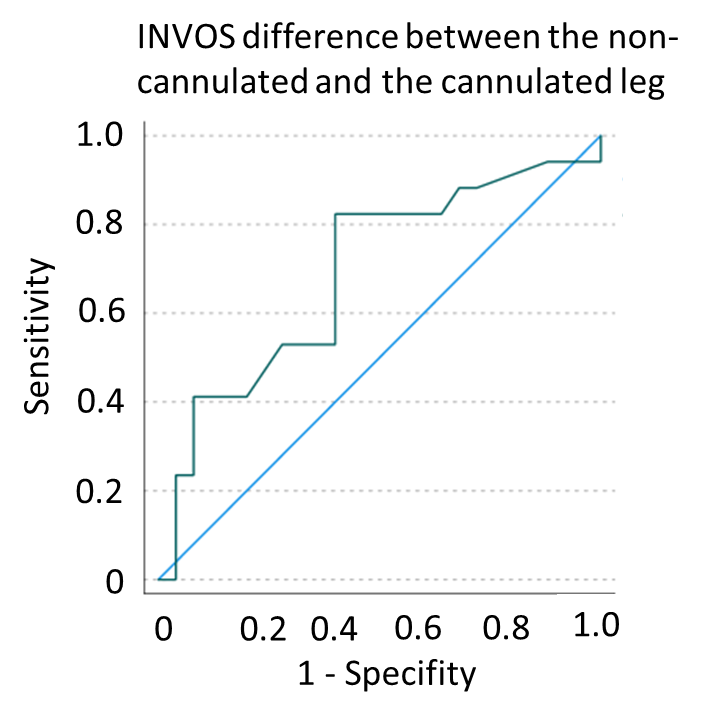


| STROBE Statement | Item No | Recommendation |
| --- | --- | --- |
| **Title and abstract** | 1 | (*a*) Indicate the study’s design with a commonly used term in the title or the abstract **[ NA ]** |
|  |  | (*b*) Provide in the abstract an informative and balanced summary of what was done and what was found **[See results section of abstract page 2 ]** |
| Introduction | | |
| Background/rationale | 2 | Explain the scientific background and rationale for the investigation being reported **[page 5 ]** |
| Objectives | 3 | State specific objectives, including any prespecified hypotheses **[ page 5 ]** |
| Methods | | |
| Study design | 4 | Present key elements of study design early in the paper **[ page 6 ]** |
| Setting | 5 | Describe the setting, locations, and relevant dates, including periods of recruitment, exposure, follow-up, and data collection **[ page 6-10 ]** |
| Participants | 6 | (*a*) Give the eligibility criteria, and the sources and methods of selection of participants. Describe methods of follow-up **[ page 6 ]** |
|  |  | (*b*) For matched studies, give matching criteria and number of exposed and unexposed **[ NA ]** |
| Variables | 7 | Clearly define all outcomes, exposures, predictors, potential confounders, and effect modifiers. Give diagnostic criteria, if applicable **[ page 7-9 ]** |
| Data sources/ measurement | 8 | For each variable of interest, give sources of data and details of methods of assessment (measurement). Describe comparability of assessment methods if there is more than one group **[ page 7-10 ]** |
| Bias | 9 | Describe any efforts to address potential sources of bias **[NA]** |
| Study size | 10 | Explain how the study size was arrived at **[ page 6-9 ]** |
| Quantitative variables | 11 | Explain how quantitative variables were handled in the analyses. If applicable, describe which groupings were chosen and why **[ page 9-10 ]** |
| Statistical methods | 12 | (*a*) Describe all statistical methods, including those used to control for confounding **[ page 9-10 ]** |
|  |  | (*b*) Describe any methods used to examine subgroups and interactions **[ NA ]** |
|  |  | (*c*) Explain how missing data were addressed **[ NA ]** |
|  |  | (*d*) If applicable, explain how loss to follow-up was addressed **[ NA]** |
|  |  | (*e*) Describe any sensitivity analyses **[ NA ]** |
| Results | | |
| Participants | 13 | (a) Report numbers of individuals at each stage of study—eg numbers potentially eligible, examined for eligibility, confirmed eligible, included in the study, completing follow-up, and analysed **[ page 11 ]** |
|  |  | (b) Give reasons for non-participation at each stage **[ page 11 ]** |
|  |  | (c) Consider use of a flow diagram **[ Figure 1 ]** |
| Descriptive data | 14 | (a) Give characteristics of study participants (eg demographic, clinical, social) and information on exposures and potential confounders **[ page 11; table 1, 3 ]** |
|  |  | (b) Indicate number of participants with missing data for each variable of interest **[table 1, 3]** |
|  |  | (c) Summarise follow-up time (eg, average and total amount) **[N/A]** |
| Outcome data | 15 | Report numbers of outcome events or summary measures over time **[ page 9-11 ]** |
| Main results | 16 | (*a*) Give unadjusted estimates and, if applicable, confounder-adjusted estimates and their precision (eg, 95% confidence interval). Make clear which confounders were adjusted for and why they were included **[ page 9-14 ]** |
|  |  | (*b*) Report category boundaries when continuous variables were categorized **[ page 9-14 ]** |
|  |  | (*c*) If relevant, consider translating estimates of relative risk into absolute risk for a meaningful time period **[N/A]** |
|  |  | Other analyses **[N/A]**  Report other analyses done—eg analyses of subgroups and interactions, and sensitivity analyses **[N/A]** |
| Discussion | | |
| Key results | 18 | Summarise key results with reference to study objectives **[ page 15 ]** |
| Limitations | 19 | Discuss limitations of the study, taking into account sources of potential bias or imprecision. Discuss both direction and magnitude of any potential bias **[ page 18 ]** |
| Interpretation | 20 | Give a cautious overall interpretation of results considering objectives, limitations, multiplicity of analyses, results from similar studies, and other relevant evidence **[ page 15-18 ]** |
| Generalisability | 21 | Discuss the generalisability (external validity) of the study results **[ page 15-18 ]** |
| Other information | | |
| Funding | 22 | Give the source of funding and the role of the funders for the present study and, if applicable, for the original study on which the present article is based **[page 31]** |
